# Importantes lésions radiologiques de spondylodiscite tuberculeuse pauci-symptomatique

**DOI:** 10.11604/pamj.2014.18.282.4982

**Published:** 2014-08-06

**Authors:** Hasina Ursèle Andrianarimanitra, Narindra Lova Hasina Ny Ony Rajaonarison, Miora Lovatiana Randrianalison, Ahmad Ahmad

**Affiliations:** 1 Service d'Imagerie Médicale et de Radiodiagnostic CHU-JRA, Antananarivo, Madagascar

**Keywords:** Tuberculose multiviscérale, spondylodiscite, imagerie, immuno-competant

## Abstract

La spondylodiscite tuberculeuse est la localisation la plus fréquente des tuberculoses ostéo-articulaires et parmi-celle-ci l'atteinte dorsolombaire est prédominante. Les formes associées à d'autres localisations de la tuberculose restent rares chez les immuno-compétants. Les auteurs rapportent une observation d'un patient présentant des signes radiologiques majeurs d'une tuberculose multiviscérale et spondylo-discale confirmée histologiquement, avec un tableau clinique pauvre afin de discuter l'intérêt de l'imagerie dans le diagnostic de cette pathologie.

## Introduction

La spondylodiscite tuberculeuse est une atteinte de corps vertébral et de disque intervertébral par le bacille tuberculeux [1]. La localisation vertébrale est la plus fréquente des tuberculoses ostéo-articulaires. Elle est grave par la possibilité d'une atteinte neurologique qui peut être importante et définitive [2]. Nous rapportons le cas d'un jeune patient, avec un tableau clinique pauvre, qui présentait des signes radiologiques majeurs de spondylodiscite tuberculeuse, confirmée histologiquement, associée à d'autres foyers de tuberculose, afin de discuter l'intérêt de l'imagerie dans le diagnostic de cette pathologie.

## Patient et observation

Il s'agissait d'un garçon de 15 ans, collégien, qui se plaignait d'une douleur basi-thoracique droite et lombaire évoluant depuis deux mois. Il a été normalement vacciné notamment avec le BCG et on ne notait pas de notion de contage tuberculeux ni de notion de tuberculose maladie antérieure. Aucune notion de ponction lombaire, ni d'acte invasive au niveau du rachis n'a été retrouvée. Le patient était en bon état général mais subfébrile à 38°C surtout le soir.

La clinique révélait une douleur à la palpation de la onzième et douzième vertèbre dorsale et de la première lombaire sans tuméfaction visible en regard. On ne notait pas de déformation de la colonne vertébrale mais il existait un syndrome pleural droit. L'examen neurologique était sans particularité notamment absence de déficit notable. La biologie était normale (globules blancs à 9 x 109/l puis 7,9 x 109/l deux semaines plus tard). CRP à 6mg/l, lymphocytes à 1,6 x 109/l soit 18%). La sérologie VIH n'a pas été faite. L'analyse biochimique de l’épanchement pleural liquidien, après ponction, a montré un liquide exsudatif avec protéine totale à 71 g/l.

**Figure 1:**
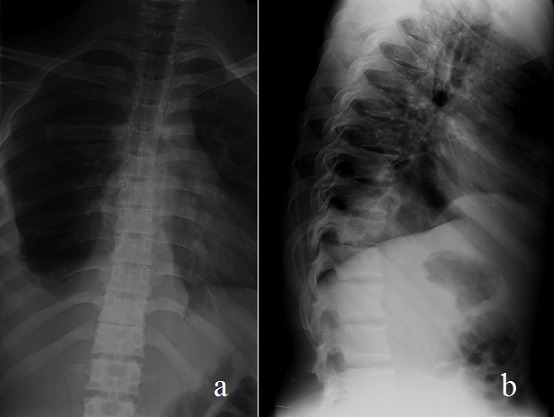
Radiographie du rachis dorsolombaire en incidence de face (a) et de profil (b) montrant un pincement de l'interligne intervertébrale T10-T11 avec destruction vertébrale en miroir, un fuseau paravertébral bilatéral et une angulation à faible courbure

La radiographie du rachis dorsolombaire montrait une ostéo-condensation de la dixième et de la onzième vertèbres dorsales (T10, T11) avec lyse en miroir et pincement de l'interligne intervertébrale correspondante avec opacités paravertébrales bilatérales correspondant à un fuseau paravertébral ainsi qu'une angulation rachidienne à faible courbure sur le profil (Figure 1).

**Figure 2:**
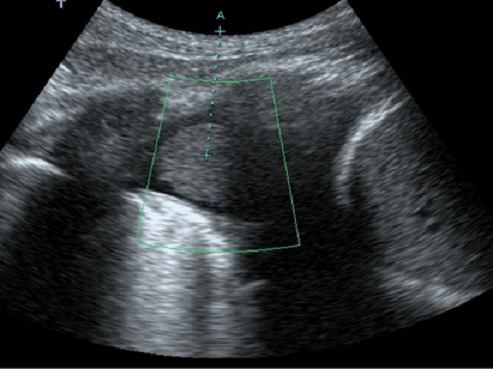
Coupe sagittale échographique passant par le cul de sac pleural droit et le dôme hépatique montrant un nodule pleural au sein d'une pleurésie

L’échographie pleurale et abdominale montrait un épanchement pleural liquidien droit enkysté, fait de liquide épais et contenant une formation échogène tissulaire (Figure 2) interne; ainsi qu'un abcès du muscle psoas gauche et affirmait le fuseau paravertébral bilatéral.

**Figure 3:**
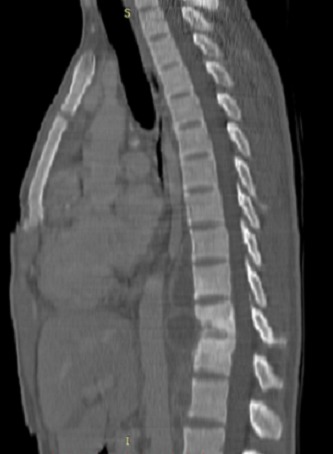
Scanner du rachis dorsolombaire en fenêtre osseuse et reconstruction sagittale montrant une destruction vertébrale en miroir au niveau T10-T11

**Figure 4:**
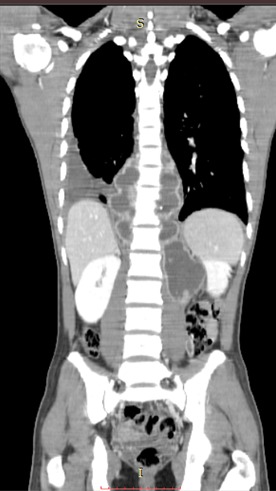
Scanner thoraco-abdomino-pelvien après injection intraveineuse de produit de contraste iodé, reconstruction coronale en fenêtre parenchymateuse montrant un fuseau d'abcès paravertébral bilatéral et un épanchement pleural liquidien droit

Le scanner thoraco-abdominal avec séquences sans et après injection de produit de contraste confirmait l'ostéolyse en miroir des plateaux de T10 et T11 et le pincement du disque correspondant avec les déformations en angulation conséquentes (Figure 3). Il a authentifié les données de la radiographie et de l’échographie en montrant la pleurésie enkystée droite avec composante tissulaire, le fuseau paravertébral et l'abcès du psoas gauche qui s’étendait jusqu'au niveau iliaque (Figure 4).

**Figure 5:**
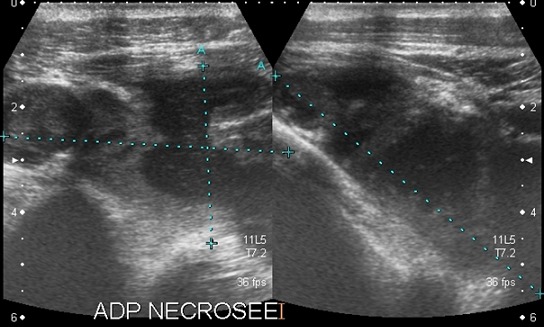
Coupes échographiques axiale et sagittale de la région axillaire gauche montrant une structure polymorphe mixte à prédominance liquidienne centrale faite de liquide épais évoquant une adénite nécrotique

L'examen histologique de pièce biopsique de la lésion tissulaire pleurale droite et l'examen anatomo-pathologique de fragments biopsiques du psoas gauche après lombotomie pour drainage de l'abcès concluaient l'origine tuberculeuse de ces lésions. L’évolution spontanée a été marquée par l'apparition de polyadénopathies superficielles et profondes d'aspect nécrotique en échographie (Figure 5) qui disparaissaient sous traitement antituberculeux seul ainsi que l'amendement des dorso-lombalgies.

## Discussion

La tuberculose ostéo-articulaire représente 3 à 5% des tuberculoses et 15% des tuberculoses extra pulmonaires [3]. La localisation vertébrale est la plus fréquente des localisations ostéo-articulaires où elle représente 35 à 55% [4]. L’âge moyen des sujets atteints de la spondylodiscite tuberculeuse est de 40 ans [1, 2]. La notion de contage tuberculeux et les signes d'imprégnation tuberculeuse ne sont pas toujours obligatoire [1].

Les rachialgies persistantes dominent le tableau clinique, puis viennent les complications neurologiques [1]. La localisation dorso-lombaire est la plus fréquente [1, 2, 5] ceci est expliquée par le fait que la dissémination de la tuberculose se fait toujours à partir d'un foyer pulmonaire [6] et c'est à partir de ce foyer initial que les bacilles vont disséminer dans l'organisme. En cas de spondylodiscite, la dissémination se fait par voie hématogène [6] via les vaisseaux issus des artères vertébrales, intercostales et lombaires. Selon la littérature, l´absence totale d'un syndrome inflammatoire biologique n´exclut pas le diagnostic de spondylodiscite tuberculeuse puisque 12 à 50% des patients atteints de cette pathologie ne présentent aucun signe biologique selon Barrière [7].

L'imagerie constitue incontestablement l'un des piliers du diagnostic du mal de Pott [2]. La radiographie standard permet de préciser le nombre de foyer atteint et les vertèbres intéressées; de montrer l'existence ou non d'un abcès cliniquement muet; d’éliminer une autre cause à l'origine de la symptomatologie; de rechercher d'autres lésions tuberculeuses (pulmonaire, ostéo-articulaire); et enfin de surveiller l’évolution des lésions [2]. La triade radiologique habituelle, comme retrouvée chez notre patient, associe un pincement discal, une destruction vertébrale avec géodes en miroir et une image d'abcès [1].

Au cours de la spondylodiscite, l’échographie tient un rôle important, car elle va permettre de rechercher des atteintes des parties molles comme les psoas et d’éventuelle localisation viscérale de la tuberculose. Cet examen permet de guider une ponction ou une biopsie [1].

La tomodensitométrie (TDM), est plus sensible que la radiographie standard dans le diagnostic de la spondylodiscite. Son intérêt est de démasquer les lésions osseuses à un stade précoce où la radiographie standard est normale [3]. Au stade de début, le disque intervertébral est le siège d'une hyperdensité évocatrice de lésion infectieuse. Les reconstructions frontales ou sagittales sont très utiles pour rechercher des érosions et des géodes sous chondrales. La TDM permet également une bonne étude des parties molles para-vertébrales à la recherche d'abcès et son extension [2]. Les séquestres osseux sont très évocateurs voire pathognomoniques de la nature tuberculeuse de la spondylodiscite, ils peuvent se présenter au sein des lésions géodiques ou au sein des abcès [2]. Ces séquestres osseux sont présents dans notre cas.

L'imagerie par résonnance magnétique (IRM), si elle est disponible, permet une étude multiplanaire plus précise et plus sensible que la TDM [8]. Elle permet d'apprécier à la fois l'atteinte osseuse, discale, épidurale, médullaire et les structures adjacentes sans oublier son apport dans le suivi post-thérapeutique [4]. Elle permet également le diagnostic différentiel avec les autres spondylodiscites infectieuses et les lésions néoplasiques [2]. Comme les données fournies par l´examen clinique et l´imagerie ne sont pas pathognomoniques, une confirmation histologique et/ou bactériologique reste nécessaire [9].

## Conclusion

La spondylodiscite tuberculeuse est la plus fréquente des tuberculoses ostéo-articulaires. Il ne faut jamais l'oublier devant une douleur rachidienne chronique qui fera demander des examens d'imagerie afin d’évoquer le diagnostic et de suivre l’évolution. La bactériologie et l'histologie permettent de donner le diagnostic de certitude.
